# Relationship between Exercise Intensity and IL-6 Increase during an 80 km Long-Distance Running Race

**DOI:** 10.3390/ijerph19116368

**Published:** 2022-05-24

**Authors:** Romain Jouffroy, Dany Anglicheau, Nicolas Mansencal, Jean François Toussaint, Juliana Antero

**Affiliations:** 1 Intensive Care Unit, Ambroise Paré Hospital, Assistance Publique-Hôpitaux de Paris (AP-HP), 92100 Boulogne-Billancourt, France; 2 IRMES—Institute for Research in Medicine and Epidemiology of Sport, INSEP, 75012 Paris, France; jean-francois.toussaint@aphp.fr (J.F.T.); juliana.antero@insep.fr (J.A.); 3 INSERM U-1018, Centre de Recherche en Epidémiologie et Santé des Populations, Paris Saclay University, 94800 Paris, France; nicolas.mansencal@aphp.fr; 4 URP 7329, Université de Paris, 75012 Paris, France; 5 Necker-Enfants Malades Institute, French National Institute of Health and Medical Research U1151, 75015 Paris, France; dany.anglicheau@aphp.fr; 6 Paris Descartes, Sorbonne Paris Cité University, 75006 Paris, France; 7 Department of Nephrology and Kidney Transplantation, Necker Hospital, Assistance Publique-Hôpitaux de Paris (AP-HP), 75015 Paris, France; 8 Department of Cardiology, Ambroise Paré Hospital, Assistance Publique-Hôpitaux de Paris (AP-HP), Centre de Référence des Cardiomyopathies et des Troubles du Rythme Cardiaque Héréditaires Ou Rares, Université de Versailles-Saint Quentin (UVSQ), 92100 Boulogne, France; 9 Centre d’Investigations en Médecine du Sport, Hôtel-Dieu, Assistance Publique-Hôpitaux de Paris (AP-HP), 75004 Paris, France

**Keywords:** running, long-distance run, exercise, intensity, interleukin 6

## Abstract

*Background*: IL-6 plasma concentration (IL-6PC) reflects the systemic inflammation related to exercise intensity level. This study aims to describe the IL-6PC kinetics during a long-distance running race. IL-6PC was measured in 20 male runners before (0 km), at each refreshment point (at 21 and 53 km, i.e., k_21_ and k_53_, respectively) and at the end of an 80 km long-distance run (k_80_). *Methods*: IL-6PC variations (absolute and relative values in each of the three sections (S)) were calculated over S1 (0_k_21_), S2 (k_21__k_53_) and S3 (k_53__k_80_) and compared with the exercise intensity (duration*race speed) within each section. *Results*: The mean IL-6PC increased during the run: 2.1 ± 0.6 ng.L^−1^ at 0 km, 21.0 ± 11.3 ng.L^−1^ at k_21_, 38.9 ± 13.0 ng.L^−1^ at k_53_ and 49.8 ± 11.9 ng.L^−1^ at k_80_. Exercise intensity increased between S1 (24.2 ± 0.5) and S2 (51.9 ± 3.2) (*p* = 0.04) but not between S2 and S3 (67.4 ± 4.5) (*p* = 0.69). IL-6PC variation was associated with exercise intensity within S1 (*p* = 0.03) and S2 (*p* = 2 × 10^−3^) and showed at least a trend within S3 (*p* = 0.06). *Conclusions*: IL-6PC increases that occur during the early stages of a long-distance run are associated with the running intensity, and then IL-6PC remain stable after the reduction in intensity related to the decrease in running speed.

## 1. Introduction

The impact of moderate and regular physical activity on the primary prevention of at least 35 chronic conditions [1] and on cardiovascular diseases has been established [2]. Its beneficial effects are partly due to the exercise anti-inflammatory effect involving biological mediators among which are interleukins (IL) [2,3].

Long-distance exercises, particularly running races, are gaining more popularity and are becoming widespread all over the world [4,5]. Initially defined as a distance over 42 km, to date a long-distance running race is defined by a duration of at least 6 h [6].

Interleukin-6 (IL-6), one of the most studied cytokines, is an inflammatory cytokine playing a central role as a mediator propagating the systemic inflammatory response. IL-6 plasma concentration is associated with and predicts the risk of future cardiovascular events [7], especially atherosclerosis [8], and events such as myocardial infarction and heart failure [9] and death [7]. Nevertheless, no causal association between IL-6 plasma concentration and illness occurrence has, to date, been clearly established.

IL-6 is released from the skeletal muscle cells into the blood related to metabolism and energy deprivation [10]. Exercise is known to increase inflammation-responsive cytokine levels, especially IL-6 [11], reaching a peak immediately after exercise [11,12,13]. The IL-6 plasma concentration increases early, within 30 min, after acute intense exercise [14]. Thereafter, there is an increase in the IL-6 plasma concentration in an almost exponential manner [15] reaching a peak at the end of the exercise before returning to pre-exercise plasma concentration levels within the next 24 h [16]. IL-6 has been particularly studied [17] during acute exercise in order to reflect on the systemic inflammation response. However, previous studies assessed pre- and post-race IL-6 plasma concentration levels [18,19,20,21] not its kinetics during the race. Factors determining IL-6 plasma concentration are mainly represented by exercise duration [12,15] and exercise intensity [12,22]. Exercise intensity may be indirectly represented by the muscle mass involved for running and/or by the speed of the race [23,24]. During a long-distance running race, because the running speed progressively decreases due to tiredness, the exercise intensity varies; consequently, the relationship between exercise intensity and IL-6 plasma concentration is not linear. To date, to the best of our knowledge, no study has reported the IL-6 kinetics during a long-distance running race.

This study aims to describe the IL-6 kinetics among runners during an 80 km long-distance running race and the relationship with exercise intensity.

## 2. Methods

Twenty amateur male participants of the 80-km Ecotrail of the Paris Ile-de-France© 2014 race (total climb of 1500 m) were prospectively included in the study. Three refreshment points were predefined by the race organisation, respectively, at 21 km, at 53 km and at 80 km. The race started at noon. Throughout the race, participants had free access to food and water.

All amateur volunteers were electronically recruited before the start of the race using an announcement on the race’s website (www.traildeparis.com, accessed on 19 May 2022) in the two weeks before the race start.

Adults (age > 18 years) of male gender and with completion of an ultra-endurance race (distance > 50 km) during the previous 12 months were included.

Those younger than 18 years old and/or female—in order to avoid the effects of unknown pregnancy—and/or those having had illness or injury in the month before the race and/or any medication or drug use in the 3 months before the race were not included in the study.

All participants gave their written informed consent for participation before the start of the race. 

Blood samples (1 mL) were collected before the race start (0 km), during the race at the end of each section (21 km and 53 km) and immediately on the finish line (80 km). Blood drawing required only a 1-min stop. Blood samples were drawn by a nurse, collected and immediately stored on ice and sent to a hospital in Paris to be analysed later. IL-6 plasma concentration was measured by the immuno-chemiluminescence method (Roche Diagnostics©, Meylan, France).

The 3 refreshment points (21, 53 and 80 km) defined 3 Sections: Section 1 (S1): 0 to 21 km (0_k_21_), Section 2 (S2): 21 to 53 km (k_21__k_53_) and Section 3 (S3): 53 to 80 km (k_53__k_80_).

In order to assess exercise intensity, absolute and relative IL-6 plasma concentration variations were calculated within each of the 3 sections and compared with the exercise intensity. The race intensity was assessed by duration*race speed, within the same sections, respectively.

Continuous variables with a normal distribution are expressed by mean ± standard deviation (SD), whereas continuous variables with a non-Gaussian distribution are expressed by median and interquartile range (1st quartile–3rd quartile). Categorical data are expressed as absolute value and percentage.

Comparisons were performed using a Wilcoxon test. Correlation was assessed with R2 Pearson correlation test. Statistical significance was defined by a *p*-value of <0.05. All analyses were performed using R 3.4.2© (http://www.R-project.org; the R Foundation for Statistical Computing, Vienna, Austria).

The race’s organization committee, an independent institutional review board, the French Committee on public safety Paris Ile-de-France IV approved the protocol (Reference: 2014/07), as did the National Heart Agency (Number EudraCT: 2014-A00205-42) on 14 March 2014.

## 3. Results

On 14 March 2020, the day of the race, the weather was clear (no rain), and the temperature was 14 °C.

All subjects had trained with a training mean time of 5 ± 3 h per week for the last 12 months, corresponding to 46 ± 18 km per week. All subjects were trail running experienced with a mean of 5 ± 3 trail running races every year and a mean of 6 ± 4 years of trail running experience. The mean age was 43 ± 7 years old, and the mean body mass index was 23.7 ± 2.3 kg.m^−2^.

The demographic characteristics of the volunteers are summarized in Table 1.

None of the subjects reported having run or played any sport in the 2 days prior to the race.

No subject declared any medication or drug use in the previous 3 months, and none reported illness within 1 month before the race.

All 20 volunteers (100%) reached the finish line, were examined and benefited from blood sample drawing before the start, at the end of each section and immediately after the end of the race. 

The mean race duration was 668 ± 60 (minimum = 549; maximum = 762) minutes.

**Figure 1:**
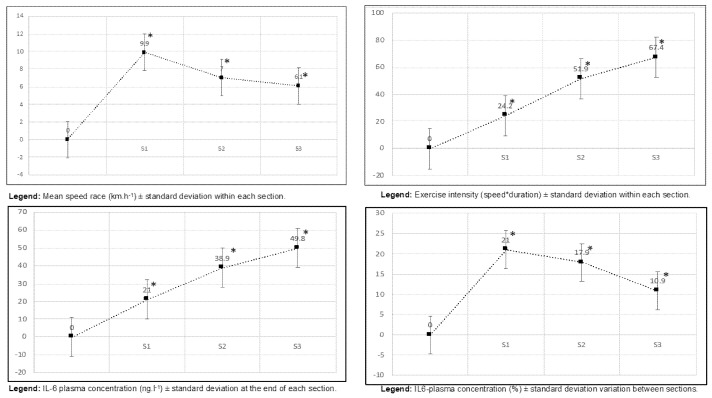
Evolution of speed race, intensity, IL_6 absolute and relative variations among the different sections. Data are expressed as mean ± SD. * means significant difference vs. S1 (*p* < 0.05).

The average race speed was 7.3 ± 0.7 (minimum = 6.3; maximum = 8.8) km.h^−1^ but progressively decreased along the three sections from S1 to S3: 9.9 ± 0.8 in S1, 7.0 ± 0.8 in S2 and 6.1 ± 0.8 km.h^−1^ in S3 (Table 1 and Figure 1).

Mean IL-6 plasma concentration significantly increased, reaching its maximal value in the last race section: 2.1 ± 0.6 ng.L^−1^ before the race, 21.0 ± 11.3 ng.L^−1^ at k_21_, 38.9 ± 13.0 ng.L^−1^ at k_53_ and 49.8 ± 11.9 ng.L^−1^ at k_80_ (Figure 1).

No significant correlation was observed between IL-6 plasma concentration and race speed and race duration (*p* > 0.05) at 21, 53 and 80 km, respectively.

Exercise intensity (speed*duration) significantly increased between S1 (24.2 ± 0.5) and S2 (51.9 ± 3.2) (*p* = 0.04) but not between S2 and S3 (67.4 ± 4.5) (*p* = 0.69) (Table 1).

IL-6 plasma concentration variation significantly differed between S1 (21.0 ± 11.3 ng.L^−1^) and S2 (17.9 ± 15.2 ng.L^−1^) (*p* = 0.01) but not between S2 and S3 (10.9 ± 13.9 ng.L^−1^) (*p* = 0.21) and S1 and S3 (*p* = 0.19).

A significant association was retrieved between exercise intensity and IL-6 plasma concentration at the end of each section: S1 (*p* = 0.03), S2 (*p* = 0.04) and S3 (*p* < 10^−3^).

IL-6 plasma concentration variation was significantly associated with exercise intensity within S1 (*p* = 0.03) and S2 (*p* = 2 × 10^−3^) but not within S3 (*p* = 0.06).

## 4. Discussion

During a long-distance running race, we observed a significant IL-6 plasma concentration increase that was significantly associated with the running race intensity. The IL-6 increase during a long running race occurs during the early stages of the race and remains stable after intensity reduction related to the race speed decreasing.

To the best of our knowledge, this study is the first to assess the evolution of IL-6 plasma concentration linked to exercise intensity.

Interleukin-6 (IL-6) is one of the most studied cytokines [15] and is an inflammatory cytokine propagating the systemic inflammatory response after being released from the skeletal muscle cells into the blood in order to meet energy deprivation [11]. Previous studies reported that after an early concentration increase [13,25], IL-6 plasma concentration reaches a peak at the end of the exercise [13] before returning to pre-exercise plasma concentration levels within the next 24 h [14]. Nevertheless, the relationship between IL-6 plasma concentration and intensity during exercise was never explored because of the methodological approaches used: previous studies assessed pre- and post-race IL-6 plasma concentration [18,19,20,21].

Exercise duration [12,15] and intensity [12,22] are the main factors determining IL-6 plasma concentration. Because during a long-distance running race the running speed progressively decreases due to tiredness, the exercise intensity also decreases, suggesting that the relationship between exercise intensity and IL-6 plasma concentration levels is not linear. We propose that the reduction in running speed mainly explains the negative relationship between IL-6 and intensity. Moreover, this assumption is supported by the constant muscle mass involved in running, explaining that the IL-6 plasma concentration level is more influenced by the speed of the race than by the race duration [23,24]. Because individual innate and acquired characteristics influence IL-6 plasma concentration at rest as well as in response to exercise [15,25,26], we chose to assess the variation between each section, each volunteer being considered as their own control. Moreover, because of the length of the race, the IL-6 plasma concentration was linked to the corresponding speed for each section.

Our results are consistent with previous studies reporting the increase in IL-6 plasma concentration with exercise duration [15,27,28,29,30] and its return to baseline after the end of the exercise [14,25,31]. For running, the ability to maintain speed depends on the muscle mass of the limbs involved [32]. The relatively lower values of plasma IL-6 concentration observed in our study compared to previous studies could be explained by the fact that for long-distance running, a smaller muscle mass seems to be more efficient, in contrast to short-distance running requiring higher explosiveness [23,24].

IL-6 is an important mediator of the inflammatory response whose exact role remains under debate between its mediation of lethal complications and as an “alarm hormone” reflecting endothelial cell injury [33].

There are strengths and limitations with the current study. This is a small sample size study. Food and drink intakes were not standardised between all volunteers and may have partly contributed to the IL-6 plasma concentration. Only male volunteers were involved in the study; thus, we cannot extrapolate the results to the female gender. We cannot exclude that the participants involved in the current study do not have an “extra-normal” level of the underlying inflammatory state and/or a moderate response [34], related to their frequent practice of long-distance exercises [35], and which would suggest their potential adaptation to the intensity of long-term running practice. We cannot exclude that participants had unknown illnesses or injuries influencing the baseline level of IL-6 plasma concentration and/or modulating the host response. Due to the study design, we are not able to determine whether the participants reached their maximal performances. The methodological design does not allow any causal conclusion between long-distance race exercise and IL-6 increase. This study reports an IL-6 plasma concentration increase without being able to determine its origin(s): blood and/or muscle and/or neuronal and/or adipocyte cells. In addition, we defined intensity by speed*race duration; nevertheless, this intensity definition has not been agreed [12,36].

Despite limitations, this study is the first to assess IL-6 plasma concentration and its variation linked to intensity during a long-distance race, observing a peak in the increase in IL-6 not reported after intensity measurement. The early IL-6 plasma concentration peak may be related to speed and we suggest that the intensity decrease reflected by the decrease in race speed may be related to a decrease in the sympathetic reserve, associated with a progressive heart rate decrease and a speed decrease, which, in long-distance racing, tends towards a limited value, depending on the maximum catabolic rate of the organism [33].

## 5. Conclusions

A significant IL-6 plasma concentration increase occurs during the early stages of a long-distance running race. This escalation remains stable after an intensity reduction related to a decrease in the race speed race. Further studies are needed to confirm these preliminary results and their significance on health.

## Figures and Tables

**Table 1:** Values of IL-6 PC, speed race within section, IL-6 plasma concentration variation between sections. Data are expressed as mean ± SD.

Parameter	S1	S2	S3
IL6-PC (ng.L^−1^) (N < 7.5)	21.0 ± 11.3	38.9 ± 13.0 *	49.8 ± 11.9 *
Speed race (km.h^−1^)	9.9 ± 0.8	7.0 ± 0.8 *	6.1 ± 0.8 *
Exercise intensity	24.2 ± 0.5	51.9 ± 3.2 *	67.4 ± 4.5 *
IL-6 PC variation between sections (ng.L^−1^)	21.0 ± 11.3	17.9 ± 15.2 *	10.9 ± 13.9

Legend: IL-6 PC = interleukin 6 plasma concentration. S1: Section 1 from 0 to 21 km, S2: Section 2 from 21 to 53 km and S3: Section 3 from 53 to 80 km. Values in brackets refer to normal range. * means significant difference vs. S1 (*p* < 0.05).

## Data Availability

Data are available on reasonable request.
